# Using Online Health Communities to Deliver Patient-Centered Care to People With Chronic Conditions

**DOI:** 10.2196/jmir.2476

**Published:** 2013-06-25

**Authors:** Martijn van der Eijk, Marjan J Faber, Johanna WM Aarts, Jan AM Kremer, Marten Munneke, Bastiaan R Bloem

**Affiliations:** ^1^Radboud University Nijmegen Medical CentreNijmegen Centre for Evidence Based PracticeDepartment of Neurology (935)NijmegenNetherlands; ^2^Radboud University Nijmegen Medical CentreScientific Institute for Quality of Healthcare (IQ healthcare)NijmegenNetherlands; ^3^Radboud University Nijmegen Medical CentreDepartment of Obstetrics and GynaecologyNijmegenNetherlands; ^4^Radboud University Nijmegen Medical CentreDonders Center for Brain, Cognition and BehaviourDepartment of Neurology (935)NijmegenNetherlands

**Keywords:** community networks, Internet, patient-centered care, long-term care, chronic disease, Parkinson disease

## Abstract

**Background:**

Our health care system faces major threats as the number of people with multiple chronic conditions rises dramatically.

**Objective:**

To study the use of Online Health Communities (OHCs) as a tool to facilitate high-quality and affordable health care for future generations.

**Methods:**

OHCs are Internet-based platforms that unite either a group of patients, a group of professionals, or a mixture of both. Members interact using modern communication technologies such as blogs, chats, forums, and wikis. We illustrate the use of OHCs for ParkinsonNet, a professional network for Parkinson disease whose participants—both patients and professionals—use various types of OHCs to deliver patient-centered care.

**Results:**

We discuss several potential applications in clinical practice. First, due to rapid advances in medical knowledge, many health professionals lack sufficient expertise to address the complex health care needs of chronic patients. OHCs can be used to share experiences, exchange knowledge, and increase disease-specific expertise. Second, current health care delivery is fragmented, as many patients acquire relationships with multiple professionals and institutions. OHCs can bridge geographical distances and enable interdisciplinary collaboration across institutions and traditional echelons. Third, chronic patients lack adequate tools to self-manage their disease. OHCs can be used to actively engage and empower patients in their health care process and to tailor care to their individual needs. Personal health communities of individual patients offer unique opportunities to store all medical information in one central place, while allowing transparent communication across all members of each patient’s health care team.

**Conclusions:**

OHCs are a powerful tool to address some of the challenges chronic care faces today. OHCs help to facilitate communication among professionals and patients and support coordination of care across traditional echelons, which does not happen spontaneously in busy practice.

## Introduction

### Background

Our health care system faces major threats. Western societies age rapidly, and as a consequence, the prevalence of people with multiple chronic conditions rises dramatically [1]. Moreover, the number of patients with complex health care needs outpaces the number of professionals with sufficient knowledge and skills to adequately care for these people [2]. Finally, health care threatens to become unaffordable due to overtreatment and costly medical advancements [3,4]. To guarantee quality and affordable health care for future generations, innovations are needed [5]. In this paper, we discuss the use of Online Health Communities (OHCs) as a tool to address some of the above challenges. We illustrate the use of OHCs for ParkinsonNet, a professional network for Parkinson disease (PD), whose participants—both patients and professionals—use various types of OHCs to deliver patient-centered care [6,7].

ParkinsonNet consists of regional allied health networks for PD in the catchment areas of Dutch hospitals. Within each network, a selected number of expert therapists are trained according to evidence-based guidelines. Neurologists are stimulated to refer PD patients to these skilled professionals. Additionally, the concept has nationwide coverage in the Netherlands with 66 regional networks and 2400 physicians, nurses, physical therapists, occupational therapists, speech-language pathologists, and dieticians involved. ParkinsonNet was developed to improve the quality of PD care delivered by allied health professionals. The implementation of ParkinsonNet has shown a profound reduction in health care utilization and costs [6]. Participants increased their PD-specific knowledge, improved the adherence to guideline recommendations, and treated a higher volume of patients per year [7].

### Online Health Communities

Platforms using social media technologies, such as Wikipedia, Facebook, LinkedIn, YouTube, and Twitter, have become extremely popular among millions of people worldwide. These tools have brought new possibilities for co-creation and communication between individuals with minimal time and cost restrictions [8]. It seems logical to apply elements of this revolution to health care. As such, social network technologies provide an Internet-based platform for communication about health and disease, for sharing care experiences, and to increase medical knowledge [9,10]. By echoing Web 2.0 principles into health care, we could help patients become active participants in their own care and more engaged partners for health professionals [11]. Moreover, Internet-based contacts are a way to expand the possibilities for communication outside the few scheduled face-to-face hospital consultations [12].

One specific example are OHCs, which consist of an Internet-based platform that unites groups of individuals with a shared goal or similar interest regardless of their whereabouts [13]. Such a group could include patients with a particular condition (eg, patients with diabetes mellitus type II), a group of professionals with a shared medical interest (eg, diabetologists), or a mixture of both patients and professionals. Members might know each other from the “real” physical world, but the strength of OHCs is their potential to connect members who would otherwise never have met because of geographical distances. Within OHCs, members interact easily using modern communication technologies such as blogs, chats, forums, and wikis (Textbox 1, an illustrative example is PatientsLikeMe, an online platform for patients with life-changing conditions who share their experiences and medical data with other patients matched for clinical conditions and geographical characteristics. This platform provides generic solutions to acquire medical information and peer support for different patient groups [14]. PatientsLikeMe is currently being used by PD patients who quantify and self-report their disease symptoms on a regular basis. These data provide health professionals with new insight into variations in symptom severity and understanding about the disease progression in PD [15].

Platforms using OHC principles are utilized by patients from various ages. Moreover, the Health and Welfare Information Portal (ZWIP) combines an electronic health record with a communication tool aiming to improve care for frail, older people. ZWIP potentially enhances patient involvement, coordination of care and collaboration among professionals [16]. Furthermore, OHCs are utilized in Dutch fertility care. Young couples gain access to their medical records containing general and personal information and communication tools with peers and their local health care team [17].

**Social media applications within an online health community.**

Blog: a blog is a series of messages published in reverse chronological order written by one of the community members (eg, scientific developments or personal care experiences).

Chat: a chat is a real-time conversation with other community members.

Forum: a forum is used for asynchronous communication with other community members (eg, patients can put questions to professionals or peers).

Library: where documents are shared with all community members (eg, information pamphlets, newsletters, scientific articles, and guidelines).

Wiki: an application within a community where all members are allowed to adapt a certain document (eg, an address list or information pamphlet).

### Open and Closed Communities

OHCs can be classified into open and closed communities based on the accessibility of the community content. The content of open OHCs can be accessed by anyone, all members are allowed to contribute to its content, and all information that is generated is openly accessible to anyone. Within a closed OHC, the content is visible to community members only. Members are allowed to make an active contribution after a community manager, that is, an individual who leads the community, has granted them access. The platform described in this paper is utilized by several patient groups in the field of PD, dermatology, stroke, MS, rheumatoid arthritis, fertility and cancer care [18].

#### Open Parkinson Communities

The Parkinson community is an open community for all people interested in PD. Members are PD patients, caregivers, and health professionals. Table 1 shows the different social media applications used within this community. Patients use the community forum for online peer support and discussions with health professionals. Often, fellow patients provide useful answers, which may alleviate the pressure on health professionals. In an open community for breast cancer patients, incorrect answers were rapidly corrected by other members [19]. A striking feature is the wiki, which, with the help of several community members, is developing into a national encyclopedia for PD.

#### Closed Parkinson Communities

**Figure 1:**
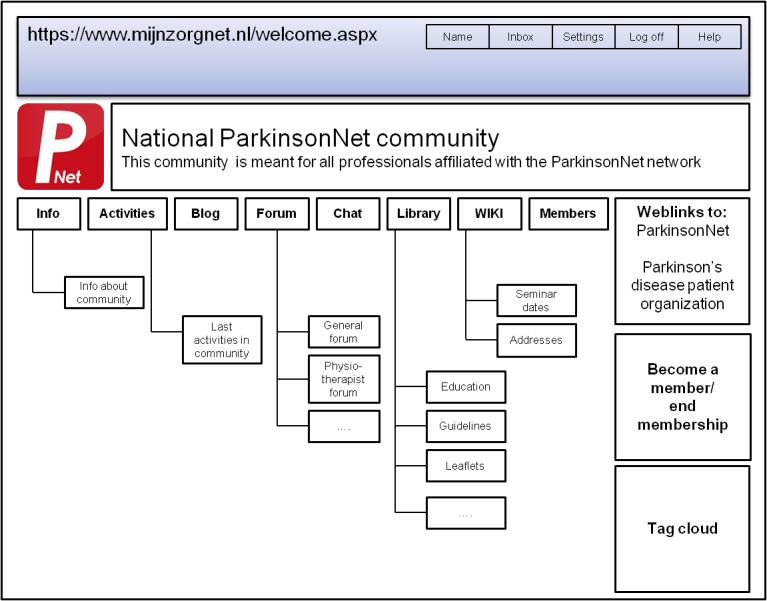
Schematic overview of the closed ParkinsonNet community.

The ParkinsonNet community is used to facilitate communication and collaboration between health professionals involved in the treatment of PD patients and is accessible to ParkinsonNet professionals only (Figure 1). After verifying the ParkinsonNet membership, the community manager grants access. The community forum has been divided into separate discussions for physical, speech, and occupational therapists, and for interdisciplinary consultation. Other applications include the community blog, where members are informed about ongoing education and guidelines; the wiki, containing an up-to-date address list of all ParkinsonNet professionals; and the community library, used for sharing presentations of multidisciplinary team meetings.

Another example is the closed community of an outpatient Parkinson clinic, which is accessible only to patients visiting the clinic and to health professionals who work there (Table 1). A distinctive feature of this community is the combination of online patient-provider and peer-to-peer communication integrated into one and the same community with both patients and health professionals from the same clinic involved. In our Parkinson center, we run such an OHC as a service to both our patients and the members of our multidisciplinary health care team. Access is restricted and controlled—to become a member, patients must first send a formal membership request. After verifying the patient identification number, the community manager grants access.

The community blog contains information about the treatment facilities that are available at our center. Within the community forum, patients are provided with facilities for communication with fellow patients and the health care team. Future patients benefit from previous discussions, which remain visible unless patients wish this to be removed. This OHC does not offer individually tailored information because the exchange of information is not private and can be seen by all members. This community type has proved to fill the gap between patients’ needs and the support our clinic can offer [18].

Some of our patients are both members of the closed outpatient Parkinson clinic and the open Parkinson community. These patients have the opportunity to ask questions on both forums. Items in the outpatient clinic are more likely to involve questions to our medical team and facts, for example, treatment options in our clinic, whereas the open national forum is more likely to contain peer contact, care experiences, and opinions.

#### Personal Health Communities for Parkinson Disease

A Personal Health Community (PHC) is a private community governed by individual PD patients. Apart from the patient, participants include one or more (ideally all) health professionals involved in the care process, and the immediate caregiver. The patient is the owner of the community and decides who is granted access to the community. The immediate caregiver can act as community manager if the patient is unwilling or unable to do so. Once gathered, the patient and the health care team exchange information and communicate about individual health problems. Like an electronic patient record, PHCs offer the opportunity to store all medical information in one central place, while allowing transparent communication across members of the health care team. Hereby, the patient is in the lead as an active and equal partner who contributes to his own health.

PHCs differ from other OHCs in two ways. First, PHC functionalities are customized and used in a different way. The blogging feature is used as a diary to inform other members about, eg, side effects of anti-Parkinson medication, the forum for online consultation of health professionals, the library to store medical information, and the wiki as a specific medical document, like a medication scheme or treatment overview. A second difference is “two-way authentication”, which adds an extra layer of security to the PHC. Patients have to enter their username, password, and a security code sent to their mobile phone.

### Active Users

The following definition of active user is applied on our platform: “The total number of users who performed at least one activity for a given day. Activities include: blog posts, blog comments, forum posts, forum replies, library uploads, library downloads, new wiki pages, wiki revisions, wiki comments, joining a group, subscribing to content or rating a post” [20]*.* Table 1 shows that over a 12-month period, 54% of the Parkinson and ParkinsonNet community members generated new content or posted a comment. Other participants may have visited the community, albeit without an active contribution.

**Table 1:** Social media applications and members of the Parkinson communities.

	Parkinson community	ParkinsonNet community	Outpatient Parkinson clinic	Personal health community
Community type	Open	Closed	Closed	Closed
Info	A national community for people interested in PD for peer-to-peer contact and patient-professional interaction	Transmural community for ParkinsonNet expert therapists for online professional interaction	Intramural community of an outpatient Parkinson clinic. Supplementary service to the existing face-to-face care relationship	A private electronic patient record of individual patients to allow personalized medicine
Members	PD patients, caregivers and health professionals 1190 (Dec. 31, 2012) 1224 (Jan. 31, 2013)	ParkinsonNet health professionals	The multidisciplinary health care team and PD patients visiting the Parkinson clinic	One PD patient, his caregiver and health professionals involved (eg physical therapist, neurologists, PCP) 147 patients 78 professionals (Jan. 31, 2013)
Active users	2012: 646 (54%) Recent month^a^: 83 (7%)	2012: 737 (54%) Recent month: 182 (13%)	2012: 56 (88%)	Data not available due to technical and privacy issues
Page views	2012: 241,093 Recent month: 24,671	2012: 76,452 Recent month: 7568	2012: 8376 Recent month: 1098	Data not available due to technical and privacy issues
Blog	Information from physicians and therapists on new trials, etiology, diagnosis and PD treatment options	Information from ParkinsonNet professionals about conferences, team meetings, new PD guidelines	Information on medication, non-motor symptoms, research in our clinic, announcement of maternity leaf local PD nurse specialist	PD patients’ diary about eg on/off fluctuations, wearing off of, side effects and daily experiences
Forum	Discussions between community members about medication, symptoms and peer support	Discussions about allied health therapy, food and diet and medication	Peer-to-peer contact between PD patients in a familiar setting and questions to the local health care team	Consultation between a patient and his professional care team about, eg, side effects
Library	Documents on allied health disciplines, driving abilities, medication and side effects, PD guidelines and scientific papers	Centralized up-to- date information: newsletters, presentations, scientific papers, PD guidelines, clinimetrics, courses	Information on treatment facilities provided by our clinic and regional peer contact	Individually tailored information, eg, physical exercises, medication schemes or a diary
Chat	No chat available	Real time conversation at assigned times between ParkinsonNet professionals	No chat available	No chat available
Wiki	A national encyclopedia for PD on diagnosis, symptoms, medication, on/off fluctuations, multidisciplinary collaboration, etiology and disease progression	Address list of all ParkinsonNet professionals, a ParkinsonNet calendar with, eg, regional ParkinsonNet meetings, conferences and symposia	No wiki available	Eg, medication or treatment overview

^a^Recent month means January 2013.

## Methods

### Implementation Strategy

Implementation of OHCs in clinical practice takes a collective effort of all health professionals involved. However, the community manager plays a vital role during the implementation and maintenance of all OHCs. The community manager of an outpatient Parkinson clinic is usually a local PD nurse specialist. PD nurse specialists are key practitioners when it comes to the coordination of care, patient education, and emotional support [21]. However, the community manager appointed in our clinic is a marketing and communication expert. The community manager distributes posters, information pamphlets, and “business cards” to patients and health professionals. The information pamphlet, which is available in every doctor’s office, contains information about the aim of the community, login procedures, and social media applications within the OHC. Other tasks of the community manager include management and maintenance of the community members’ database, generation of content, motivation of health professionals and patients to participate, and monitoring of the expert forum. Recently, the first 10 PD patients received training on navigation through the online outpatient clinic.

The community manager of the ParkinsonNet community is a marketing and communication expert as well. She visited all 66 regional ParkinsonNet networks to educate health professionals about OHCs. ParkinsonNet professionals are urged to enroll in the ParkinsonNet community as part of their membership. Currently, the ParkinsonNet community is the main source of information for the professionals in the network. Some information about new guidelines and state-of-art courses can be found only within this community.

In 2011, we introduced the Personal Health Community (PHC) in four Parkinson clinics in the Netherlands. During regular home visits, patients learn to navigate through and utilize the PHC by a local PD nurse specialist. ParkinsonNet organizes workshops to engage patients and health professionals in the pilot regions. Additionally, an information pamphlet, poster and a video to promote the PHC were introduced. Roughly, implementation of PHCs includes three phases: a pilot phase concerning patients and professionals from the Parkinson clinic only, a second phase in which primary care providers are invited, and a third phase in which new clinics are included. Our first experiences show that PHCs facilitate emotional support, health care accessibility, and improve relationships between professionals and patients.

## Results

### The Added Value of OHCs in Chronic Care

Based on our first experiences in PD care and the international literature, OHCs have four major advantages to improve the quality of chronic care. These include facilitation for the exchange of medical experience and knowledge, enhancing interdisciplinary collaboration across institutions and traditional echelons, providing a platform to support self-management, and the ability to improve patient-centered care.

### OHCs Facilitate the Exchange of Medical Experience and Knowledge

Due to rapid advances in medical knowledge, many health professionals lack specific expertise and experience to address the complex health care needs of chronic patients [1,2]. Therefore, health care is increasingly organized within specialized networks, like ParkinsonNet [22,23]. Professional networks enhance information exchange, facilitate communication among participants and foster the adoption of new knowledge, such as revised guidelines [24,25]. Traditionally, these network processes occur largely offline during physical encounters, such as medical conferences. However, with the advent of modern communication technologies, professional networks can now be supported online. Within OHCs, professionals connect and communicate more easily, regardless of their working place within the network, and regardless of time. Moreover, OHCs can be used to develop disease-specific expertise among all community members, patients, and professionals, interested in a particular chronic condition [26].

### OHCs Enhance Interdisciplinary Collaboration Across Institutions and Traditional Echelons

Health care delivery can become fragmented for chronic patients when they acquire relationships with multiple professionals and institutions. Increasingly, chronic care has evolved from individual consultations into multidisciplinary teamwork with care given by various physicians and therapists, who often work in different departments or organizations [27]. To manage complex patients with multiple co-morbidities, health professionals must collaborate to make coordinated decisions and share responsibilities in health outcomes [28]. Yet, the collaboration and coordination of care should be improved considerably [29]. Given their synchronous and asynchronous communication capacity, OHCs offer a platform for supporting medical decision-making and interdisciplinary collaboration across professionals caring for complex patients [26,30,31]. OHCs enable communication between community members who are not able to have face-to-face interaction at any point in time. Moreover, OHCs bridge geographical distances and enable interaction across institutions and traditional echelons. An example is the Canadian Virtual Hospice, with information and support on palliative and end-of-life care [32]. Patients, close family members, and caregivers interact in several peer-to-peer discussion forums or private messages with a team of palliative care experts. Normally, these interactions would not have been possible due to physical limitations and geographical distances.

### OHCs Provide a Platform to Support Self-Management

Typically, patients have a passive role and lack the tools to self-manage their condition [33]. However, modern patients search the Internet for medical information, wish to have open communication channels with their physicians, and prefer to participate in making treatment decisions [34]. Self-management refers to the efforts to enhance patient participation and assisting patients to gain control over their lives [35]. The concept is associated with improved communication between patients and clinicians, and it enhances quality of life [36]. Supporting patients with chronic diseases like type 2 diabetes, arthritis, and asthma to self-manage their condition helps to improve the quality and safety of care and reduces costly and inappropriate use of health care resources [37,38]. Increasingly, the Internet is used to support self-management and actively engage patients in treatment decisions [39]. Chronic patients using online communication tools become more knowledgeable, feel better socially supported and empowered, and have improved behavioral and clinical outcomes compared to nonusers [40,41]. Examples that include OHC principles are patient participation in online peer support groups and access to PHCs [15,42]. PHCs allow patients to have access to medical records, control their own online information, and enable individualized health communication [43].

### OHCs Have the Ability to Improve Patient-Centered Care

Patient-centeredness is defined as “providing care that is responsive to individual patient preferences, needs, and values, and ensuring that patient values guide all clinical decisions*”* [44]. Contrary to some perceptions, patient-centeredness is not just about being nice to patients, but engaging them to become active participants in their care [45]. The concept is known for its advantages in terms of reduced health care utilization and improved efficiency, patient-doctor communication, treatment compliance, and health outcomes [46-48]. OHCs enhance patient-centered care by improved access to personalized information, emotional support, and patient participation [15-42,49]. PHCs are essentially patient-centered, while they engage patients in their care process and tailor care to their individual needs. Professionals have the opportunity to benefit from patient peer-to-peer conversations that take place in OHCs by knowing that they have more effectively addressed their patients’ needs [50]. Blog and forum items often involve aspects of patient-centered care, such as information and emotional support needs, patients’ willingness to participate in treatment decisions, or an experienced lack of continuity of care.

## Discussion

### Barriers for Implementation in Clinical Practice

Why do only a few innovations become part of routine practice and why do most fail to survive beyond the pilot phase? To answer this question, full understanding of the clinical and technological barriers and incentives for achieving behavior change in practice is needed [51,52]. The following barriers to Internet technologies may be at play.

First, the implementation of Internet innovations can radically affect health care delivery and professionals’ daily work processes, requiring considerable time and willingness to learn [53]. Doctors may be hesitant to adopt technologies that imply an interruption of their traditional practice patterns. The requirement of additional time is a prominent barrier to physician technology acceptance [54].

Second, the implementation of OHCs into clinical practice demands a paradigm shift in control and power, out of the hands of those who deliver care, into the hands of those who receive it [13]. Professionals should no longer regard patients as passive objects, but rather as equal, participatory partners who contribute to their own health. Thus, OHCs require a change in the mindset of both professionals and patients. Not surprisingly, in the age of Facebook, young clinicians may struggle to maintain professional distance on one hand and have close, meaningful relationships with their patients on the other [55].

Third, besides behavioral change, safety and financial issues have to be solved [56]. To ensure a safe and secure environment, the Dutch government authorizes PD patients to apply their personal verification code, normally used to complete and submit a tax return form to the tax authority, while using our OHC platform. Health professionals are allowed to access closed communities only via their unique, electronic identity. Additionally, for OHCs to become integrated into everyday use, new and viable business models are needed. To utilize OHCs in daily practice is time consuming; however, they may also substitute normal ways of care delivery. Generally, health care is reimbursed by face-to-face interactions and offline medical services. Bearing this in mind, we would like to introduce the term “blended health”, analogous to blended learning, which combines face-to-face contact with the possibilities of online tools. The intended result is a health care system not driven by technology, but using technology as a tool to facilitate patient-centered, collaborative care.

More and more, innovations in health care are based on Internet technologies and the willingness of PD patients to participate in such interventions is growing [57]. Generally, health related Internet use is associated with age and level of education [58]. The European Union investigated the level of Internet access within the 27 member states. Household Internet access ranged from 45% in Bulgaria to 94% in the Netherlands [59]. Therefore, Internet access is assumed to be a minor limitation in the adoption of OHCs in the Netherlands.

### Conclusions

OHCs are a powerful tool to address some of the challenges chronic care faces today. A challenge now is to perform an in-depth evaluation of our platform, which is simultaneously being designed, developed, and deployed [60]. Further evaluation should address user needs, risks, benefits, and cost implications before OHCs can be fully adopted in daily practice [61,62]. We expect that innovations like OHCs can help to facilitate high-quality and affordable health care for future generations. Chronic care demands an integrated approach tailored to the needs of individual patients to optimize outcomes. In the absence of a formal team structure, OHCs help to facilitate communication among professionals and patients and support coordination of care across traditional echelons, which does not happen spontaneously in a busy practice.

## Abbreviations

OHC: online health community
PD: Parkinson disease
PHC: personal health record
